# High Levels of DegU-P Activate an Esat-6-Like Secretion System in *Bacillus subtilis*

**DOI:** 10.1371/journal.pone.0067840

**Published:** 2013-07-04

**Authors:** Catarina Baptista, Hugo Condessa Barreto, Carlos São-José

**Affiliations:** Centro de Patogénese Molecular – Unidade de Retrovírus e Infecções Associadas (CPM-URIA), Faculdade de Farmácia da Universidade de Lisboa, Lisboa, Portugal; Centre National de la Recherche Scientifique, Aix-Marseille Université, France

## Abstract

The recently discovered Type VII/Esat-6 secretion systems seem to be widespread among bacteria of the phyla *Actinobacteria* and *Firmicutes*. In some species they play an important role in pathogenic interactions with eukaryotic hosts. Several studies have predicted that the locus *yukEDCByueBC* of the non-pathogenic, Gram-positive bacterium *Bacillus subtilis* would encode an Esat-6-like secretion system (Ess). We provide here for the first time evidences for the functioning of this secretion pathway in an undomesticated *B. subtilis* strain. We show that YukE, a small protein with the typical features of the secretion substrates from the WXG100 superfamily is actively secreted to culture media. YukE secretion depends on intact y*ukDCByueBC* genes, whose products share sequence or structural homology with known components of the *S. aureus* Ess. Biochemical characterization of YukE indicates that it exists as a dimer both *in vitro* and *in vivo*. We also show that the *B. subtilis* Ess essentially operates in late stationary growth phase in absolute dependence of phosphorylated DegU, the response regulator of the two-component system DegS-DegU. We present possible reasons that eventually have precluded the study of this secretion system in the *B. subtilis* laboratory strain 168.

## Introduction

Bacteria are equipped with several protein secretion systems that allow them to survive and modulate the interactions with the different environments they encounter [1]. These systems are fundamental in processes such as cell differentiation, horizontal gene transfer, nutrients uptake and, in the context of bacterial infection, in the establishment of pathogenic interactions with eukaryotic cells [2]. Recent studies have uncovered a new secretion system, particularly prevalent among *Actinobacteria* and *Firmicutes*, which has been called Type VII secretion system (T7SS) and Esat-6-like secretion system (Ess), respectively [3], [4]. The single and more general designation WXG100 secretion system (Wss) has been proposed as a better suited nomenclature for this secretion pathway [1], [5].

Initial clues for a T7SS came from the *in silico* analysis of the *Mycobacterium tuberculosis* virulence effectors ESAT-6 (early secreted antigenic target, 6 kDa) and CFP-10 (culture filtrate protein, 10 kDa), encoded by *esxA* and *esxB* genes, respectively, which were known to be secreted despite not having any recognizable secretion signal [6], [7]. Distant homologues of ESAT-6/CFP-10 were identified in Gram-positive bacteria, all sharing a central WXG motif and a length of *ca.* 100 amino acids. Actually, all those proteins appear to belong to the WXG100 superfamily (pfam06013) [8]. WXG100 coding genes were found to cluster with genes for membrane proteins, ATPases and/or chaperones, leading to the proposal that these could form an apparatus that secreted WXG100 proteins [6], [7], [8]. Interestingly, the predicted ATPases belonged to the FtsK/SpoIIIE family, which are translocases involved in chromosome segregation during cell division or endospore differentiation [9]. It was speculated that WXG100 protein secretion would thus rely on ATP hydrolysis [8].

Numerous subsequent studies have lent experimental support to the existence of this secretion pathway, and several genes clustering with those of WXG100 proteins were demonstrated to be essential for the functioning of the system (see [3] for a review). Most importantly, T7S-like systems have been shown to play an important role in the virulence of important human pathogens like *M*. *tuberculosis* and *Staphylococcus aureus* [4], [10], [11], [12], [13], [14]. The *M. tuberculosis* genome encodes five T7SS, most commonly designated by ESX-1 to -5 [15], whereas *S. aureus* has only one Esat-6-like secretion system, the Ess [4].

**Figure 1:**
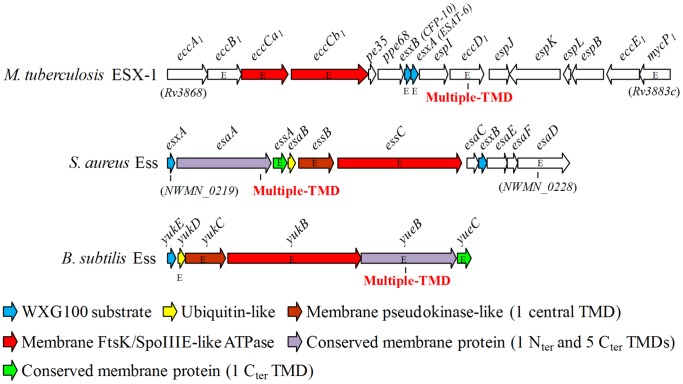
Schematic representation of the gene clusters encoding core components and substrates of T7S-like systems in *M. tuberculosis* (ESX-1), *S. aureus* (Ess) and *B. subtilis* (Ess). The nomenclature of ESX-1 genes is that proposed by Bitter et al [15]. Note that, for simplicity, several ESX-1 secretion-associated protein (e*sp*) genes, located immediately or far upstream of *M. tuberculosis* e*ccA1*, are not represented. Genes consistently described as essential (or important) for secretion of cognate WXG100 proteins, or of other specific substrates, are marked with the letter “E” [3], [4], [16], [17] (see text for *B. subtilis* Ess). Genes coding for products sharing conserved domains are depicted with the same color code, whereas those specific of each system are colored in white. Features of conserved gene products are indicated below (TMD stands for transmembrane domain). EssB and YukC harbor a pseudokinase domain [18], [19].

WXG100 secretion substrates and the proteins with FtsK/SpoIIIE domains are the only conserved elements that can be found between the T7S-like systems of the broad range of *Firmicutes* and *Actinobacteria*. However, within each taxa there is conservation of several proteins of this pathway [3], [5]. In *Firmicutes* these are prototyped by some Esa and Ess proteins of the *S. aureus* Ess (Figure 1) [4], [10]. As noticed in previous studies [3], [4], [8], the Gram-positive model bacterium *B. subtilis* has at least one WXG100 protein, YukE, which clusters with homologues of *S. aureus* Esa/Ess proteins (Figure 1).

Some T7S-like systems do not seem to confer any obvious advantage during *in vivo* growth of pathogenic bacteria [20], [21], while in other they mediate DNA transfer by a conjugation-like mechanism [22]. Thus, the study of the putative *B. subtilis* Esat-6-like secretion system (BsEss) might reveal new cellular roles for this export pathway and contribute to the understanding of its evolution. In this work we present the first experimental evidences supporting Ess functioning in *B. subtilis*. We identify key components of the system and provide important insights on the regulation of its activity.

## Materials and Methods

### *B. subtilis* Strains, Phage and Growth Conditions

*B. subtilis* strains (Table 1) were pre-cultured overnight in LB medium [26] at 30°C with aeration. The next day cultures were diluted 100-fold in fresh medium and grown at 37°C, with agitation, until the indicated growth phase. When required, erythromycin, chloramphenicol, neomycin, IPTG and xylose were used at 0.5 µg/ml, 5 µg/ml, 7.5 µg/ml, 1 mM and 0.5% concentrations, respectively. Phage SPP1 titration was as described previously [27].

**Table 1:** *B. subtilis* strains used in this work.

Strains	Genotype or relevant features	Reference/Source
L16601	*B. subtilis* 168	[23]
ATCC 6051	*B. subtilis* subsp *subtilis*, wild type isolate	ATCC*
168*degU32*(Hy)	L16601 derivative, *degU32*(Hy), cm^R^	This study
W654	ATCC 6051 derivative, *degU32*(Hy), cm^R^	[24]
W648	ATCC 6051 derivative, *degS* (non-sense)::pCA191, cm^R^	[24]
WTF28	ATCC 6051 derivative, *degU::cat*, cm^R^	[25]
W654*yukE*	W654 derivative, *yukE*ΩpCBM6, ery^R^ cm^R^	This study
W654*yukD*	W654 derivative, *yukD*ΩpCBM7, ery^R^ cm^R^	This study
W654*yukC*	W654 derivative, *yukC*ΩpCBM8; ery^R^ cm^R^	This study
W654*yukB*	W654 derivative, *yukB*ΩpCBM9, ery^R^ cm^R^	This study
W654*yueB*	W654 derivative, *yueB*ΩpCBM10, ery^R^ cm^R^	This study
W654*yueC*	W654 derivative, *yueC*ΩpCBM11, ery^R^ cm^R^	This study
W654*yueB P_xylA_-yueB*	W654.*yueB* derivative, *amyE*:: *P_xylA_-yueB,* ery^R^ cm^R^ neo^R^	This study

* American Type Culture Collection.

### Construction of *B. subtilis* Mutants

Extraction of *B. subtilis* chromosomal DNA was as reported previously [27]. Development of competence and transformation of *B. subtilis* strains was according to Yasbin et al [28]. pMutin4-disrupted genes *yukE*, *yukD*, *yukC*, *yukB*, *yueB* and *yueC* were transferred to *B. subtilis* W654 by transforming this strain with chromosomal DNA from *B. subtilis* 168 derivative strains CBM6, CBM7, CBM8, CBM9, CBM10 and CBM11 [27], respectively. The same method was used to transfer the *degU32*(Hy) allele present in strain W654 genome to the *B. subtilis* 168 strain L16601, yielding 168*degU32*(Hy), and to transfer the *amyE::P_xylA_-yueB-neo^R^* cassette from strain CSJ6 [27] to strain W654*yueB*, yielding W654*yueB P_xylA_-yueB*. Transformants were selected for their appropriate antibiotic resistance and genome structures confirmed by PCR. The presence of the *degU32*(Hy) mutation (H_12_L) [29] was confirmed by DNA sequencing.

### Purification of YukE and Production of Anti-YukE Antibodies

The coding sequence of YukE (complementary to the sequence encompassing coordinates 3276141 to 3276434, Acc. N0. NC_000964.3) was PCR amplified using the primer pair KE-Nco/KE-Xma (sequences provided upon request), carrying *Nco*I and *Xma*I restriction sites, respectively, and ligated to the expression vector pIVEX3.2d (Roche Applied Sciences) after digestion of both molecules with *Nco*I and *Xma*I. This vector is designed to drive the expression of cloned genes under the control of phage T7 φ10 promoter and to allow the production of the corresponding proteins C-terminally fused to a hexahistidine tag. The recombinant plasmid was first recovered in strain XL1-Blue MRF’ (Stratagene) and, after confirmation of sequence correctness by DNA sequencing, it was transferred to the expression *Escherichia coli* strain CG61. This strain is a BL21 derivative (Stratagene) that overproduces phage T7 RNA polymerase upon temperate upshift [30]. CG61 cells carrying pIVEX2.3d::*yukE* were selected at 28 °C in medium supplemented with ampicillin (100 µg/ml) and kanamycin (40 µg/ml).

Expression conditions of YukE-His_6_ and production of total protein extracts were as described previously for protein YueB780 [31]. YukE-His_6_ was purified from cleared extracts by affinity chromatography using a HisTrap™ HP column (GE Healthcare) coupled to an AKTA-Prime system (GE Healthcare). The column and elution buffers had the same composition of the lysis buffer (50 mM HEPES-Na pH 7.0, 300 mM NaCl, 50 mM imidazole), except that the imidazole concentration in the elution buffer was 500 mM. YukE-His_6_ was eluted from the column in a single step with elution buffer.

YukE-His_6_ recovered from the affinity step was further purified by size exclusion chromatography in a HiPrep 16/60 Sephacryl S-300 HR column (GE Healthcare) equilibrated in YukE buffer (50 mM HEPES-Na pH 7.0, 300 mM NaCl) and run at a flow rate of 0.5 ml/min. Pure YukE-His_6_ was kept at 4°C for short periods or at –80°C for long term storage. The Stokes radius derived from the *K_av_* value of soluble YukE-His_6_ was determined by extrapolation from a plot of Stokes radii of standard proteins *versus* (−log*K_av_*)^1/2^ [32]. The column void volume (*V_0_*) was determined with blue dextran 2000 (GE Healthcare). The standard proteins (Bio-Rad) were thyroglobulin (molecular mass  = 670 kDa; Stokes radius  = 8.6 nm), γ-globulin (158 kDa; 4.8 nm), ovalbumin (44 kDa; 2.73 nm) and myoglobin (17 kDa; 2.08 nm) [32].

A rabbit polyclonal anti-serum was raised against purified YukE-His_6_ (service purchased to ACIVET, FMV-UTL, Lisbon).

### Production of Protein Extracts and Western Blot Analysis

Fifty milliliter samples of *B. subtilis* cultures were collected for preparation of protein extracts when growth reached the end of exponential phase (T0) or two hours after entry in stationary phase (T2). Strain W654 derivatives with individual knockouts of BsEss genes were grown in presence of 1 mM IPTG to guarantee expression downstream of each inactivated gene, except for strain W654*yueC*. This strain is a *yueC* conditional mutant, that is, *yueC* is silent or expressed in the absence or presence of IPTG, respectively [27]. The samples were centrifuged for cell recovery and the supernatants filtrated through 0.2 µm membranes to eliminate the remaining cells.

Proteins from cell-free supernatants were precipitated with an equal volume of PRMM solution (0.05 mM pyrogallol red, 0.16 mM sodium molybdate, 1.0 mM sodium oxalate, 50.0 mM succinic acid, 20% [vol/vol] methanol in H_2_O, adjusted to pH 2.0 with HCl), essentially as described by Caldwell and Lattemann [33]. The protein precipitate was solubilized in buffer containing 50 mM Tris.Cl pH 8.8, 7 M urea and 2 M thiourea. The cellular pellet was washed with 50 mM HEPES-Na pH 7, 150 mM NaCl and then resuspended in 1.5 ml of lysis buffer (50 mM HEPES-Na pH7, 300 mM NaCl) supplemented with protease inhibitor cocktail (Complete EDTA-free, Roche Applied Science). Cells were disrupted by performing 10 bursts of 1 min (60% power, 0.5-pulse) in a sonicator (Vibra Cell MS2T, Sonic Materials), with pauses of 1 min between each burst. Samples were kept on ice during the whole process to avoid overheating. Gross insoluble material from total cellular extracts was eliminated by centrifugation (10,000g, 20 min, 4°C) and the supernatant (cytoplasm plus membrane fraction) recovered.

Protein quantification was performed by the Bradford method (Bio-Rad Laboratories). After SDS-PAGE, gels were either stained with Coomassie blue to monitor the quality and relative protein quantities or transferred to 0.2 µm nitrocellulose membranes (Bio-Rad) for Western blot analyses. Rabbit polyclonal antibodies raised against pure YukE-His_6_, YueB780 [31] and TrxA-His_6_ [34] were used for immunodetection at 1∶10,000, 1∶30,000 and 1∶5,000 dilutions, respectively. Antigen/antibody complexes were detected with the Chemioluminescence Western Blotting Kit (Roche Applied Science), using Luminata^TM^Forte (Western HRP substrate, Millipore) for the chemiluminescence reaction when detecting YukE.

### YukE Cross-linking Experiments

The cross-linking agent BS^3^ (Sigma-Aldrich) was used for YukE cross-linking both *in vitro* and *in vivo*. The 20 mM working solution of the cross-linking agent was prepared in water immediately before use. For the *in vitro* cross-links this solution was further diluted 2-fold in 2X YukE buffer (see above) and then two concentration sets of pure YukE-His_6_ (set-1: from 0.4 to 10.3 µM; set-2: from 20 to 84 µM) were incubated with 0.25 and 1 mM BS^3^, respectively, during 30 min at room temperature. For the *in vivo* cross-links, 20 ml of cell-free supernatant from a W654 culture at stage T2 (see above) were 100-fold concentrated using a Vivaspin20 filtration unit (5,000 MWCO, PES, Sartorius Stedim). The concentrate was dialyzed against 1L of 25 mM HEPES pH7, 250 mM NaCl using a Slide-A-Lyser® Dialysis Cassete (7,000 MWCO, ThermoScientific) and the protein content estimated by the Bradford method. A total of 2.5µg protein was incubated with two concentrations of BS^3^ (1 mM and 5 mM), during 30 min at room temperature. All reactions were quenched with 50 mM Tris.Cl pH 7.5 and the cross-linking products separated in 15% SDS-PAGE. *In vitro* reactions from set-1 and the *in vivo* reactions were analyzed by Western blot using anti-YukE-His_6_ antibodies, whereas those from set-2 were visualized by Coomassie blue staining. Control reactions without the cross-linking agent were similarly prepared.

### Bioinformatics Analysis

Protein homology searches were carried out with BLASTP [35] using the NCBI’s nonredundant protein sequence database. Protein conserved domains were predicted with NCBI’s tool CDD [36]. Multiple protein sequence alignments were performed with ClustalW2 [37]. The Phyre^2^ web software was used for prediction of protein 3D structures [38].

## Results

### Bioinformatics of the *B. subtilis* Ess

The gene cluster encoding the putative *B. subtilis* Ess, here referred to as *BsEss*, comprises genes *yukEDCByueBC* (Figure 1). Recent determinations of growth condition-dependent transcriptomes [39] indicate that *BsEss* should work as an operon whose transcription is initiated by a SigA-dependent promoter upstream of *yukE*, although this gene seems also to have an independent transcriptional and/or post-transcriptional regulation, as suggested by previous studies [27], [40].

The deduced products of *BsEss* share sequence homology and/or predicted structural features with known substrates, components or proteins associated with the two best studied T7S-like systems, the ESX-1 of *M. tuberculosis* and the Ess of *S. aureus* (Figure 1). The FtsK/SpoIIIE-like ATPase, a core component that is believed to power secretion is encoded by *yukB*. The ubiquitin-like protein YukD [41], the YukC protein with pseudokinase-like fold [18], [19] and the membrane proteins YueB and YueC are homologous to *S. aureus* Ess elements and are conserved in putative T7S-like systems of other *Firmicutes*, such as *Streptococcus agalactiae* and *Listeria monocytogenes* [3], [42]. YueB is the membrane receptor essential for phage SPP1 infection [27], [31].

The array and synteny of genes composing *BsEss* is absolutely conserved in other *Bacillus* species like *B. amyloliquefaciens* DSM 7 (Acc. N0. NC_014551), *B. licheniformis* DSM 13 (Acc. N0. NC_006322), *B. pumilus* SAFR-032 (Acc. N0. NC_009848) and *B. megaterium* DSM 319 (Acc. N0. NC_014103). Interestingly, and as noticed previously [10], [17], the genetic makeup of the T7S-like systems in the pathogenic species *B. thuringiensis* (*e.g.* Acc. N0. NC_005957) and *B. cereus* (*e.g.* Acc. N0. CP001176) closely follows that of the *S. aureus* Ess. The *B. anthracis* T7S-like system is very dissimilar to the *B. subtilis* and *S. aureus* Ess systems [43].

**Figure 2:**
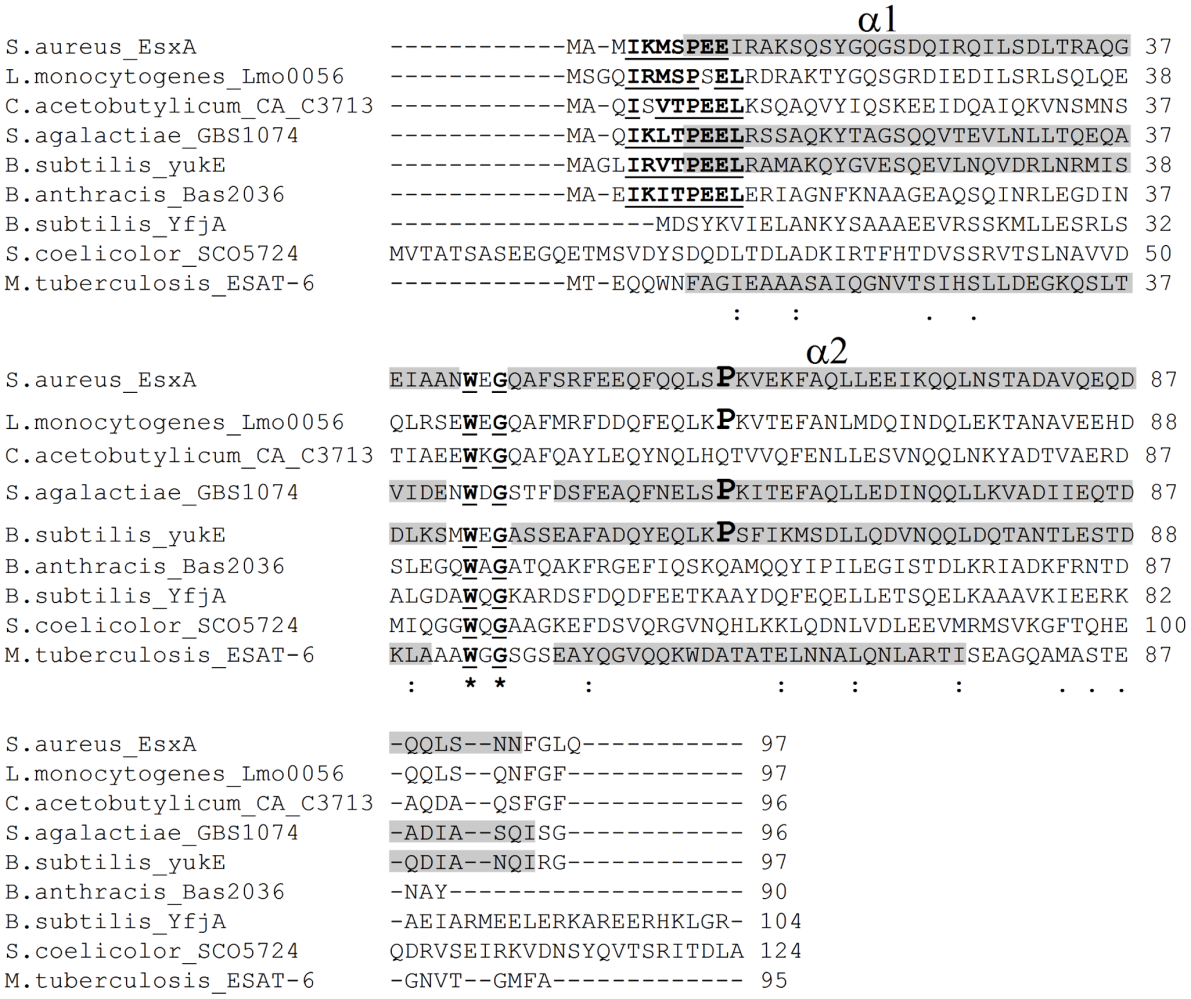
Multiple sequence alignment of representative WXG100 proteins from *Actinobacteria* and *Firmicutes*. The absolutely conserved W and G residues forming the WXG signature are in bold and underlined. WXG is part of the loop that in the tridimensional structures of the subunits EsxA, GBS1074 and ESAT-6 connects the two antiparallel α-helices α1 and α2 (formed by the gray-shaded residues) [44], [45], [46]. YukE segments predicted to form the α1 and α2 helices are also indicated by gray shading. The presence of a proline residue (boldface and enlarged font) in helix α2 was proposed to be a signature of WXG100 homodimer formation [45]. The consensus sequence I-K/R-M/V/L-S/T-P-E-E-L, highlighted in the top 6 sequences, is conserved in a large subset of YukE closest homologues from *Firmicutes*, with residues I and P being absolutely conserved (not shown). Note that *B. subtilis* YfjA is a distant homologue of YukE. Asterisk, fully conserved residues; colon, conservation of residues with strongly similar properties; period, conservation of residues with weakly similar properties.

As already mentioned, WXG100 proteins are common substrates of T7S-like systems. In general, one or two WXG100-coding genes cluster with core genes of the system (Figure 1) [3], [8], but additional WXG100 proteins can be encoded from distant loci [43]. The best candidate substrates of the *B. subtilis* Ess were identified previously [8] and are encoded by *yukE*, the first gene of the *BsEss* cluster (Figure 1) and *yfjA*, which lies in a different locus. Both YukE and YfjA exhibit the signature (WXG motif) and length (about 100 residues) characteristic of the ESAT-6/WXG100 protein superfamily (Figure 2) [8]. In this work we have probed the operation of the *B. subtilis* Ess by studying secretion of YukE.

### YukE Stable Production and Secretion Depends on DegU∼P

Most of the studied WXG100 proteins are secreted to bacterial culture media. Several components of T7S-like systems have been identified simply based on gene mutations that inhibit this secretion [3], [47]. We have followed a similar approach and thus studied YukE secretion by the classical lab strain *B. subtilis* 168 when grown in LB medium. We failed though to detect significant amounts of YukE both in cellular extracts and culture supernatants, independently of the bacterial growth phase and despite the ability of our polyclonal serum to detect low quantities of the purified, recombinant YukE (see below).

The negative results with strain 168 prompted us to search for alternative growth conditions and/or genetic backgrounds that could favor expression/functioning of the BsEss. Interestingly, at least three genome-wide transcriptomics/proteomics studies [24], [48], [49] suggested that *BsEss* is part of the DegS-DegU regulon and that its expression is activated by the phosphorylated form of DegU (DegU∼P). DegS and DegU are, respectively, the sensor kinase and the response regulator of a two component system regulating important post-exponential-phase processes in *B. subtilis*, including genetic competence, cell motility, biofilm formation and production of degradative enzymes and of poly-γ-glutamic acid [50], [51], [52]. Also relevant in this respect were the reports on the marked phenotypic differences between lab strain 168 and some undomesticated strains, particularly the fact that several Deg-regulated processes such as swarming motility, biofilm formation and exoprotease production are partially inhibited in domesticated strains [24], [53], [54], [55].

Since according to the literature expression of *BsEss* genes would be positively regulated by DegU∼P, we have hypothesized that functioning of BsEss could be attenuated in strain 168, as observed for other DegU∼P-dependent processes. To test this we have studied YukE production and secretion in the undomesticated *B. subtilis* strain ATCC 6051 (virtually identical to strain NCBI 3610) [56], here referred to as WT strain, where a direct link between DegU∼P and expression of some *BsEss* genes was previously established [24].

**Figure 3:**
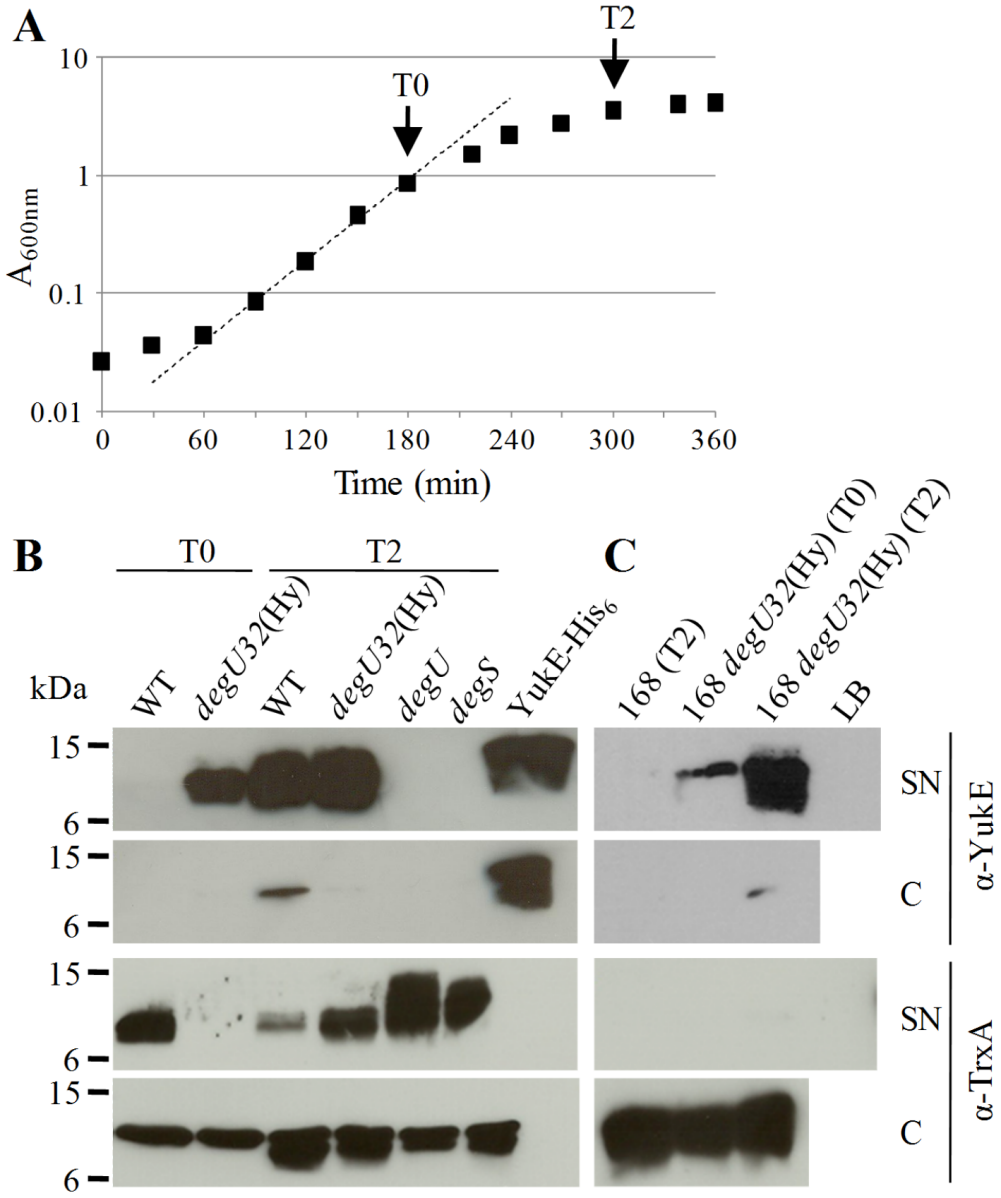
YukE production and secretion by *B. subtilis* strains ATCC 6051 (WT) and 168 and by their derivatives carrying mutations in *deg* genes. A. Growth curve of WT strain in LB at 37°C. A fitting curve for the exponential growth phase is presented. Growth curves of *deg* strains showed no significant differences (not shown). The arrows indicate the time points where culture samples were collected for production of protein extracts. T0, end of exponential growth phase; T2, 2 hours after entry in stationary growth phase. B. Immunodetection of YukE and TrxA (cytosolic control protein) in supernatant precipitates (SN) and cell extracts (C) prepared from T0 and/or T2 culture samples of strain ATCC 6051 (WT) and its derivatives W654 (*degU32*(Hy)), WTF28 (*degU*) and W648 (*degS*). Each lane was loaded with 20 µg total protein, except that of pure YukE-His_6_ (100 ng)_._ C. Immunodetection of YukE and TrxA in SN and C fractions prepared from T0 and/or T2 culture samples of strain 168 and its *degU32*(Hy)) derivative. The loaded protein amounts were as in panel B, except for the LB precipitate control that was with a volume equivalent to the maximum loaded SN fraction. The corresponding Coomassie blue stained gels of SN and C extracts are provided as Figure S1.

Culture samples of the WT strain collected in late exponential growth or 2 hours after entry in stationary phase (Figure 3(A)) were processed for precipitation of total proteins present in cell-free supernatants (SN fraction) and for preparation of cell protein extracts (C fraction). In these new conditions we could clearly detect YukE in the SN fraction of stationary growth phase cultures, but not in that of exponentially growing cultures (Figure 3(B)). The much stronger signal of YukE in the SN fraction when compared to the C fraction indicated that it accumulated in the culture medium, but not in cells.

We have also observed a DegU∼P positive regulation on YukE production and secretion. In clear difference to the WT strain, YukE could be easily detected in the supernatant of either exponential or stationary phase cultures of an ATCC 6051 derivative carrying the mutation *degU32*(Hy) (strain W654), although with a stronger signal still observed in stationary phase (Figure 3(B)). This mutation increases the half-life of the phosphorylated form of DegU [29], thus leading to an augmented expression of genes activated by DegU∼P [48]. None of the protein fractions prepared from ATCC 6051 derivatives carrying inactivated *degU* or *degS* genes revealed the presence of YukE, irrespective of the growth phase (Figure 3(B)). Moreover, the transfer of the *degU32*(Hy) allele to strain 168 was sufficient to partially restore YukE secretion and accumulation in culture supernatants (Figure 3(C)).

We have tried to perform the typical loading controls of SN fractions by using antibodies against known secreted proteins. However, perhaps due to a pleiotropic effect of *deg* mutations on secretion, we could not obtain consistent results. Yet, the corresponding Coomassie blue-stained gels of SN and C fractions of Figure 3 showed that, within each type of protein extract (SN or C), there were no major differences between the protein amounts loaded per lane (Figure S1). The Bradford quantification method seemed to give overestimates of protein content in SN samples, probably due to the colored PRMM precipitates (see methods), and thus SN lanes had systematically less protein than the corresponding C lanes (compare panels A and B of Figure S1). We have also observed some variation in the protein band profiles of SN extracts when comparing WT and *deg* mutants (Figure S1(A)). Analysis of the protein extracts with antibodies specific for a protein that normally resides in the cytoplasm (TrxA) [34] indicated that accumulation of YukE in culture supernatants did not correlate with increased cell lysis (Figure 3(B,C)).

**Figure 4:**
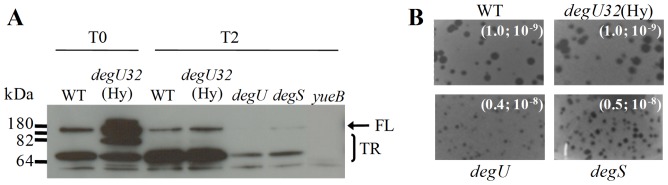
YueB production and phage SPP1 plating in the undomesticated *B. subtilis* strain ATCC 6051 (WT) and its *deg* mutants. A. Western blot analysis of YueB polypeptides in cell extracts of the WT and derivative strains carrying the mutations *degU32*(Hy), *degU* and *degS*. The protein extracts were prepared from T0 and/or T2 samples as in Figure 3. Lane “*yueB*” is a control extract prepared from a *yueB* mutant strain to help identify the immune reactive species that are YueB specific. FL and TR indicate the position of the full length and truncated polypeptides of YueB, respectively (absent in “*yueB*” lane). Each lane was loaded with 20 µg total protein. B. Phage SPP1 efficiency of plating (EoP) in *deg* strains. The EoP value and the phage dilution (10^−8^ or 10^−9^) that produced each image are indicated in parenthesis. Note the reduced plaque size in *degU* and *degS* mutants.

We have also evaluated the impact of growth phase and of *deg* mutations on the production of the membrane protein YueB, a putative component of BsEss. Previous studies have shown that YueB is mainly detected in Western blots as truncated polypeptides, suggesting that it undergoes proteolytic processing [27], [57]. The predominant species is a *ca*. 65 kDa band doublet, which is only resolved in long runs of 8% polyacrylamide gels. The full-length YueB and another truncated product of about 75 kDa are typically detected with a relatively weak signal. We observed much lower levels of the full-length and truncated YueB forms in the *degU* and *degS* mutants, when compared to those obtained in the WT and *degU32*(Hy) strain (Figure 4(A)). This diminished YueB production should be responsible for the deficient phage SPP1 plating phenotype observed in the *degU* and *degS* strains (Figure 4(B)). Interestingly, the *degU32*(Hy) mutation seemed to produce higher accumulation of the YueB in exponential rather than in stationary growth phase (Figure 4(A)). A similar observation was reported by Mäder et al [48] for several genes of the *BsEss* cluster.

In conclusion, the results indicated that YukE stable production and secretion was absolutely dependent on high levels of the phosphorylated form of DegU and that stabilized DegU∼P (in *degU32*(Hy) strain) might also increase production of the putative BsEss component YueB during late exponential growth. Based on these observations, all subsequent studies of YukE secretion and BsEss functioning were performed in the *degU32*(Hy) genetic background (strain W654), with cultures grown to late exponential growth phase.

### The *BsEss* Gene Cluster is Involved in YukE Secretion

To study the role of the *BsEss* locus on YukE production and secretion we took advantage of an available collection of *B. subtilis* 168 mutants carrying pMutin4-based, individual *BsEss* gene disruptions [27]. The advantage of performing gene disruptions through integration of pMutin4 derivatives is that, in principle, only the target gene is affected. This is achieved by two main properties of the vector [58]: i) vector-encoded transcriptional terminators block transcription initiated upstream of the integration site, and ii) the vector-borne, IPTG-inducible promoter Pspac allows expression downstream of the inactivated gene, thus bypassing polar effects in case of operon structures.

**Figure 5:**
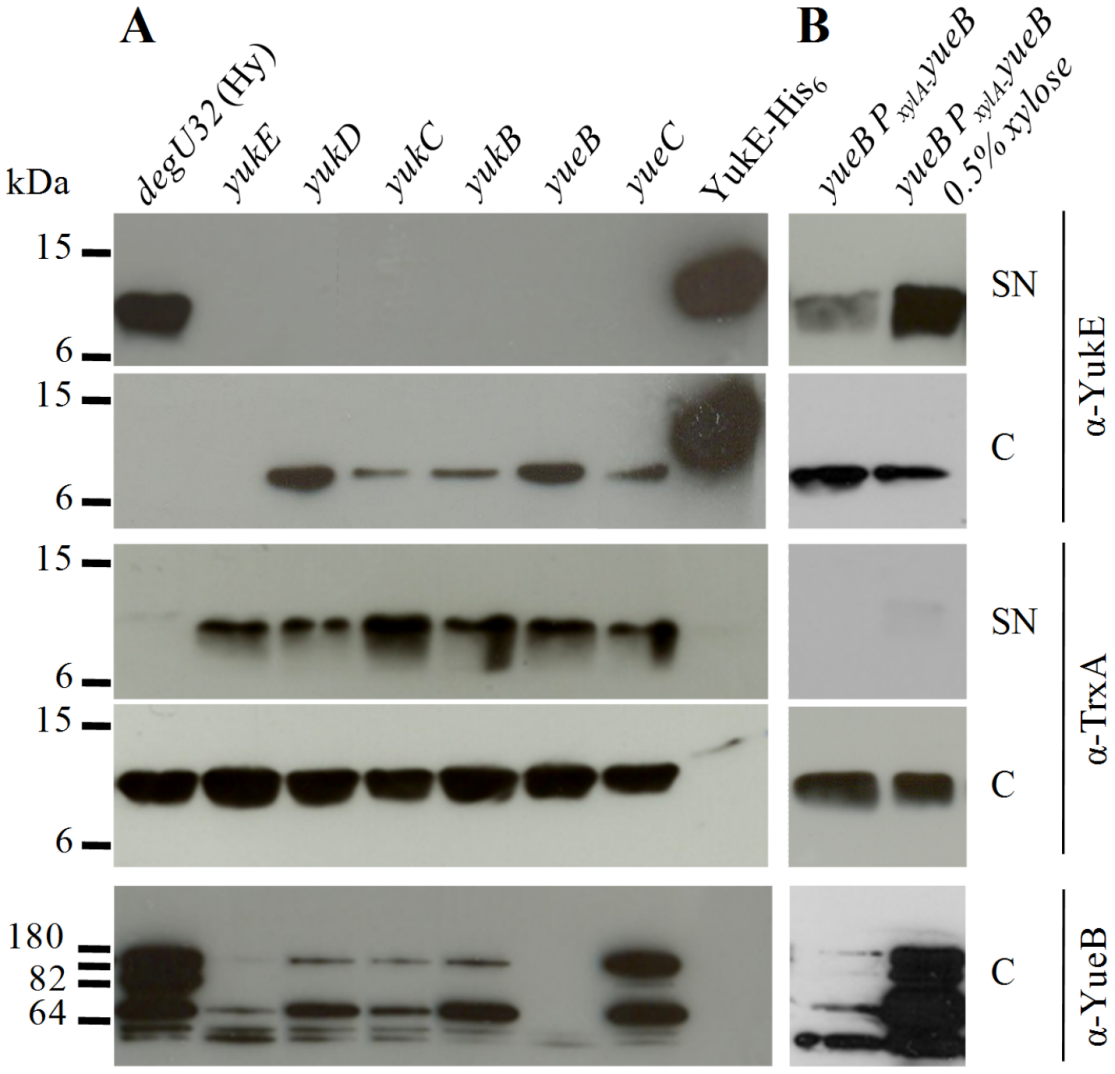
YukE production and secretion by *BsEss* mutant strains. A. YukE and TrxA (cytosolic control protein) were immunodetected in supernatant precipitates (SN) and cell protein extracts (C) of strain W654 (*degU32*(Hy)) and its derivatives with individual knockouts of *BsEss* genes. Note that the YukE signal shown for cell extracts results from an overexposed film when compared to that of SN fractions. Immunodetection of YueB polypeptides is also shown for cell extracts. Each lane was loaded with 20 µg total protein, except that of the control that was loaded with 100 ng of pure YukE-His_6_. B. Immunodetection of YukE and TrxA in SN and C fractions of the *yueB* complementation strain, in absence or presence of 0.5% xylose. The immunodetection of YueB polypeptides and the amount of loaded protein was as in panel B. The corresponding Coomassie blue stained gels of SN and C extracts are provided as Figure S2.

After transferring each *BsEss* gene disruption to the W654 strain, we have studied YukE production/secretion as described in the previous section. The results showed that integrants *yukD* to *yueC* were blocked in YukE secretion (Figure 5(A)). Interestingly, cells from these strains accumulated some YukE, something that was not observed with the control strain W654. However, the amount of cell-accumulated YukE was much lower than that buildup in the supernatant of the control strain, suggesting that YukE is unstable within cells. Similar results were reported for EsxA/EsxB of *S. aureus* when key elements of Ess were inactivated [4]. YukE could not be detected in any of the protein fractions produced from the *yukE* integrant, as expected. Analysis of the SN fractions with anti-TrxA antibodies revealed that the level of cell lysis during culture growth did not varied significantly among the different tested strains (Figure 5(A)).

The properties of the pMutin4 strategy (see above) were globally confirmed in a previous study for the specific case of the 168 *BsEss* mutants, where we have shown that polar effects on *yueB* expression resulting from integrations upstream this gene could be essentially bypassed by adding IPTG to the culture media [27]. However, since the results of Figure 5(A) were obtained in a different genetic background, we have complemented at least one mutation (*yueB*) and have monitored YueB production in all mutants for correct interpretation of the results.

The *yueB* mutation was complemented by inserting a xylose-induclible copy of the gene in the dispensable *amyE* locus of strain W654*yueB*. In presence of the inducer YukE secretion was restored, confirming the role of YueB in this secretion pathway (Figure 5(B)). Unexpectedly, YueB accumulation seemed to be decreased in all integrants but *yueC*, most obviously when considering the full-length YueB polypeptide (see α-YueB in Figure 5(A)). The YueB level was particularly diminished in the *yukE* integrant. These results suggested that pMutin4 integrations upstream *yueB* affected the expression of this gene, even in presence of IPTG. Nevertheless, this lower YueB accumulation was not sufficient to explain the lack of YukE secretion in mutants *yukD* to *yukB*, as we could detect some secretion of YukE in the *yueB* complementation strain even in absence of xylose. This indicated that the very low levels of YueB resulting from P_xylA_ leaky expression were sufficient to support secretion (Figure 5(B)). Coomassie blue-stained gels of the extracts analyzed in Figure 5 are provided as *Supplemental material* to show the even loading of SN and C extracts (Figure S2).

In summary, the results indicated that the presence of YukD to YueC products is necessary for YukE secretion and accumulation in the extracellular medium.

### YukE is a Dimer in vitro and in vivo

Structural studies of the best known substrates of T7S-like systems, the WXG100 proteins, revealed that some cognate pairs form heterodimers, as it happens with ESAT-6/CFP-10 of *M. tuberculosis* [44], [59], whereas other members of this protein family exist as homodimers, as shown for *S. aureus* EsxA (*Sa*EsxA) and *S. agalactiae* GBS1074 [45], [46]. When isolated in solution, ESAT-6 is a monomeric, globule-like protein, with about 75% content of helical secondary structure, whereas CFP-10 forms an unstructured, random coil and monomeric polypeptide. However, when mixed these two proteins tightly associate to form a heterodimeric complex with each subunit adopting a helix-loop-helix fold [44], [59]. The *Sa*EsxA and GBS1074 subunits adopt a similar topology, with two (α1 and α2) side-by-side, antiparallel helices folded to create an elongated elliptical cylinder [45].

**Figure 6:**
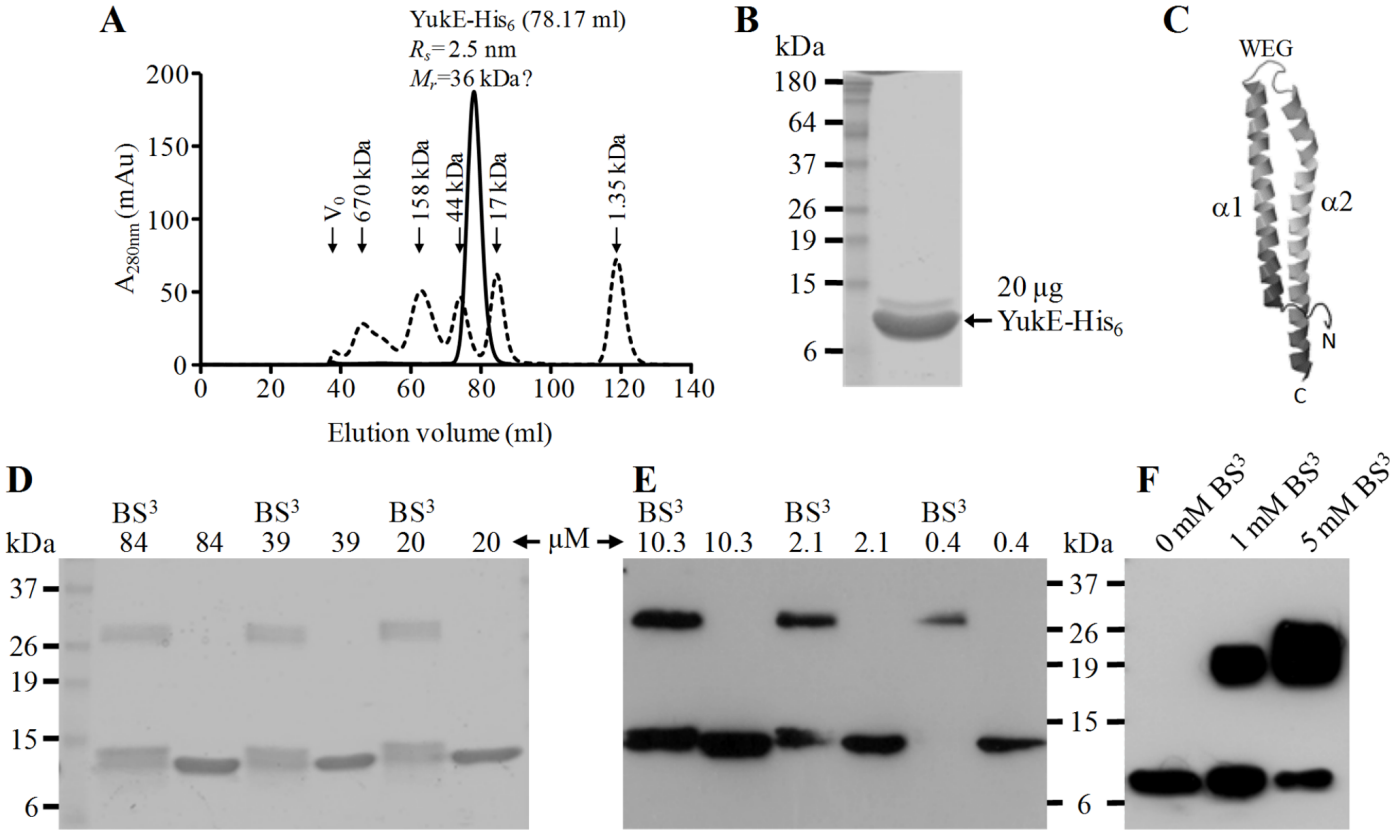
Oligomeric state of YukE. A. Purified YukE-His_6_ from the affinity chromatography step was loaded into a size exclusion column (see methods) and the eluted material was continuously monitored by taking absorbance measurements at 280 nm (A_280 nm_). The elution volume (ml) of the YukE-His_6_ peak (solid curve) and the derived Stokes radius (*R_s_*) and relative mass (*M_r_*) are indicated. Note that the mass was estimated assuming that YukE-His_6_ has the same hydrodynamic properties of the globular polypeptides composing the protein standard (dashed curve). The column void volume (*V_0_*), the elution volume of standard proteins and their corresponding masses are also indicated. B. SDS-PAGE and Coomassie blue staining of YukE-His_6_ from the peak fraction of the size exclusion chromatography. C. Modeling of the YukE subunit 3D structure by Phyre^2^ (97% YukE sequence coverage, 99.8% confidence, template = GBS1074). D,E. *In vitro* cross-linking of YukE. Two concentration sets of purified YukE-His_6_, from 84 to 20 µM (D) and from 10.3 to 0.4 µM (E) were incubated in the presence or absence of the cross-linking agent BS^3^. After SDS-PAGE separation (D, 2 µg protein per lane; E, 50 ng protein per lane), the cross-linking products were revealed by Coomassie blue staining (D) or by immunodetection with anti-YukE-His_6_ antibodies (E). F. *In vivo* YukE cross-linking. YukE present in a concentrated culture supernatant of strain W654 (2.5 µg total protein, see methods for details) was cross-linked in presence of 1 or 5 mM BS^3^ and the cross-linked products detected with anti-YukE-His_6_ as in panel E.

To gain insight on the oligomeric state of YukE, we have overproduced in *E. coli* a recombinant form of the protein C-terminally tagged with a hexahistidine tail (YukE-His_6_, 12.17 kDa). We obtained highly pure fractions of recombinant YukE (Figure 6(B)) after a purification procedure that involved a metal affinity chromatography, followed by size exclusion chromatography (Figure 6(A)). YukE-His_6_ eluted from the size exclusion column as single symmetrical peak, with an estimated Stokes radius of 2.5 nm, which would correspond to a relative molecular mass of about 36 kDa if YukE behaved as a globular polypeptide like those composing the protein standard (Figure 6(A)). This hydrodynamic radius and apparent mass suggested that YukE-His_6_ oligomerized and/or formed elongated structures [60].

To further probe the oligomeric state of recombinant YukE, a range of decreasing protein concentrations (from 80 to 0.4 µM) was treated with the cross-linking agent BS^3^, followed by analysis of the cross-linking products by SDS-PAGE. The results (Figure 6(D,E)) revealed a single cross-linking product, independently of the protein concentration, with an average mass derived from its electrophoretic mobility of 28 kDa. This mass is *ca* 2.2-fold higher than the apparent mass of the YukE-His_6_ monomer (13 kDa). Thus, the results strongly suggest that YukE-His_6_ is a dimer in solution, similarly to the WXG100 proteins *Sa*EsxA and GBS1074 [45], [46]. Most importantly, when we treated native YukE present in the supernatant of strain W654 with the same cross-linking agent we have also observed the appearance of a single, dimeric cross-linking product (Figure 6(F); note that the predicted mass of native YukE dimer is ∼22 kDa).

Thus, the results indicated that YukE accumulates as a dimer in the extracellular environment. Interestingly, the predicted α2 helix of YukE shows a conserved proline (Pro_61_) that was proposed to be a signature of WXG100 homodimer formation (Pro_60_ in *Sa*EsxA and GBS1074, Figure 2) [45]. Submission of YukE to the Phyre^2^ server for 3D structure prediction [38] returned a model for the subunit (Figure 6(C)), where 97% of YukE primary sequence was modeled with 99.8% confidence when using GBS1074 as template. The model reveals the expected helix-loop-helix fold, with the WXG signature lying in the loop and with helices α1 and α2 adopting an antiparallel configuration.

## Discussion

Several *in silico* studies have identified *B. subtilis* YukE as a WXG100 protein, which could be secreted by an Esat-6-like secretion system encoded by the gene cluster *BsEss* (Figure 1). However, functioning of this protein export pathway was never demonstrated in this bacterial species. In this work we have confirmed previous data from genome-wide expression studies that linked *BsEss* to the DegS-DegU regulon [24], [48], [49]. In fact, our results indicate that not only *BsEss* expression, but also YukE stable production and secretion require high levels of DegU∼P, which build up in cells upon transition to stationary growth phase. This could be at least one of the reasons why BsEss functioning was never demonstrated in the classic *B. subtilis* 168, since this strain is known to be impaired in DegU∼P-dependent processes. Previous proteomics studies with *B. subtilis* 168 grown to exponential or stationary growth phases failed to unambiguously detect YukE extracellularly [61], [62].

*B. subtilis* 168 has accumulated during its domestication several mutations that seem to diminish DegU phosphorylation or its action as transcriptional activator, namely mutations blocking *degQ* and *swrA* expression. DegQ was shown to enhance phosphotransfer from DegS∼P to DegU and, of note, to stimulate transcription of *degU* and *yukC* [24]. On the other hand, Ogura and Tsukahara [54] demonstrated that SwrA seems to stabilize the binding of DegU to the promoter of *ycdA*, a gene involved in swarming motility, increasing its expression. None of these mutations are present in stain ATCC 6051 [63], where we could demonstrate BsEss functioning. However, YukE secretion was only partially restored when we transferred the *degU32*(Hy) allele to strain 168 (Figure 3(C)), suggesting that this strain might carry other defects affecting BsEss activity.

Under suitable genetic and growth conditions YukE is actively secreted and accumulates in the extracellular media. The *in silico* analysis of YukE primary sequence and the YukE cross-linking experiments performed *in vitro* and *in vivo* strongly suggest that this WXG100 secretion substrate is exported as a homodimer, with each subunit adopting a fold essentially identical to that known for *Sa*EsxA and GBS1074 [45], [46].

YukE stable production, secretion and accumulation in culture supernatants depended on intact *yukDCByueBC* genes. Based on their conservation among several Gram-positive bacteria and given their particular sequence relatedness to studied elements of the *S. aureus* Ess, it is likely that the referred genes encode regulators and components of the secretion apparatus that assembles in the *B. subtilis* cell envelope. Interestingly, one of the products necessary for YukE secretion, YueB, was recently shown to preferentially accumulate near or at cell poles [57]. The same subcellular localization was observed for elements of the mycobacteria ESX-1 T7SS [64], [65], some of which should form a membrane complex as observed for mycobacteria ESX-5 [66].

The precise contribution of each BsEss element to YukE secretion is presently unknown. Sequence and structural data of YukD- and YukC-like proteins led to the proposal that these proteins may work as assembly factors and/or structural components of the secretion machinery [18], [19], [41], [67]. The YukD protein family includes *S. aureus* EsaB (Figure 1). Interestingly, transposon inactivation of *esaB* produced no effect on EsxA/EsxB secretion [4], whereas our *yukD* mutant could not secret YukE. EsaB was shown to negatively regulate production of EsaC and EsaD (Figure 1), but not of EsxA and EsxB. EsaC is an *S. aureus* specific substrate that is also secreted by the Ess pathway, whereas EsaD is a membrane protein required for efficient secretion of EsxA [10], [17]. Recently, the *S. aureus* YukC-equivalent EssB (Figure 1), a membrane protein which is also essential for secretion [4] was shown to limit accumulation of EsaB and EsaD [68]. The reason why inactivation of the Ess equivalents YukD and EsaB produce such distinct secretion phenotypes is presently an intriguing question.

We also obtained results divergent from those reported for the *S. aureus* Ess when we disrupted *yueB*. The absence of YueB abolished YukE secretion, whereas inactivation of its Ess equivalent, EsaA (Figure 1), produced no effect on EsxA/EsxB export [4]. The genomes of *B. subtilis* and *S. aureus* encode YueB and EsaA paralogues that exhibit the same predicted conserved domains [27]; these are YhgE (BSU10160) for *B. subtilis* and NWMN_2542 and NWMN_2276 in *S. aureus* strain Newman. It may happen that, in contrast to YhgE, NWMN_2542 and/or NWMN_2276 can compensate the lack of EsaA function.

The remaining two genes of the *BsEss* gene cluster, *yukB* and *yueC*, revealed to be essential for YukE secretion, similarly to what happens with their *S. aureus* counterparts *essC* and *essA*, respectively (Figure 1) [4]. YukB, EssC and the *M. tuberculosis* ESX-1 components EccCa_1_/EccCb_1_ (Figure 1) harbor one or more FtsK/SpoIIIE-like domains. Secretion of WXG100 proteins in *S. aureus* requires at least one of these domains [4], whose predicted ATPase activity is proposed to fuel the secretion pathway [8]. All tested FtsK/SpoIIIE-like proteins associated to these systems have been shown to be essential for secretion, except EssC of *B. anthracis*. An explanation for this unexpected result may reside on the fact that *B. anthracis* encodes three additional paralogues, besides the actual SpoIIIE acting during sporulation, which might replace the EssC function [43]. Thus far, no obvious role has been proposed for the predicted membrane protein EssA (YueC in *B. subtilis*), despite being essential for Ess functioning in *S. aureus* [4].

One obvious question that emerges from this work relates to the cellular function of the BsEss. Our results indicate that this secretion system is activated upon entry in stationary growth phase and that this activation depends on high levels of DegU∼P. High amounts of DegU∼P are known to build up in a subpopulation of cells when *B. subtilis* enters its stationary phase cell differentiation program [51], [69]. Thus, it is conceivable that activation of BsEss in a population subset may benefit the entire cell community by secreting WXG100 proteins and eventually other proteins/factors. In fact, it has been suggested that WXG100 proteins might function as adaptors and/or chaperones that assist secretion of the “actual” effector proteins [22], [45]. Now that we have established suitable conditions for BsEss expression and functioning, it will be interesting to determine the exoproteome of the system and to study in which cell subpopulation(s) BsEss is more active, aiming to understand its biological function. Our work also opens new venues to study the yet poorly understood molecular mode of action of T7S-like systems.

## Supporting Information

Figure S1 Coomassie blue-stained gels of the SN (A) and C (B) extracts subjected to Western blot analysis in Figure 3(B,C) (see text for details). Lane “LB” in panel A is to show the contribution of LB proteins to SN fractions.
(TIF)

Figure S2 Coomassie blue-stained gels of the SN (A) and C (B) extracts subjected to Western blot analysis in Figure 5(A,B) (see text for details).
(TIF)
