# Supplementary figures and images for: High Levels of DegU-P Activate an Esat-6-Like Secretion System in Bacillus subtilis
Source: PLoS One. 2013 Jul 4;8(7):e67840. doi: 10.1371/journal.pone.0067840 (PMC3701619; doi:10.1371/journal.pone.0067840)

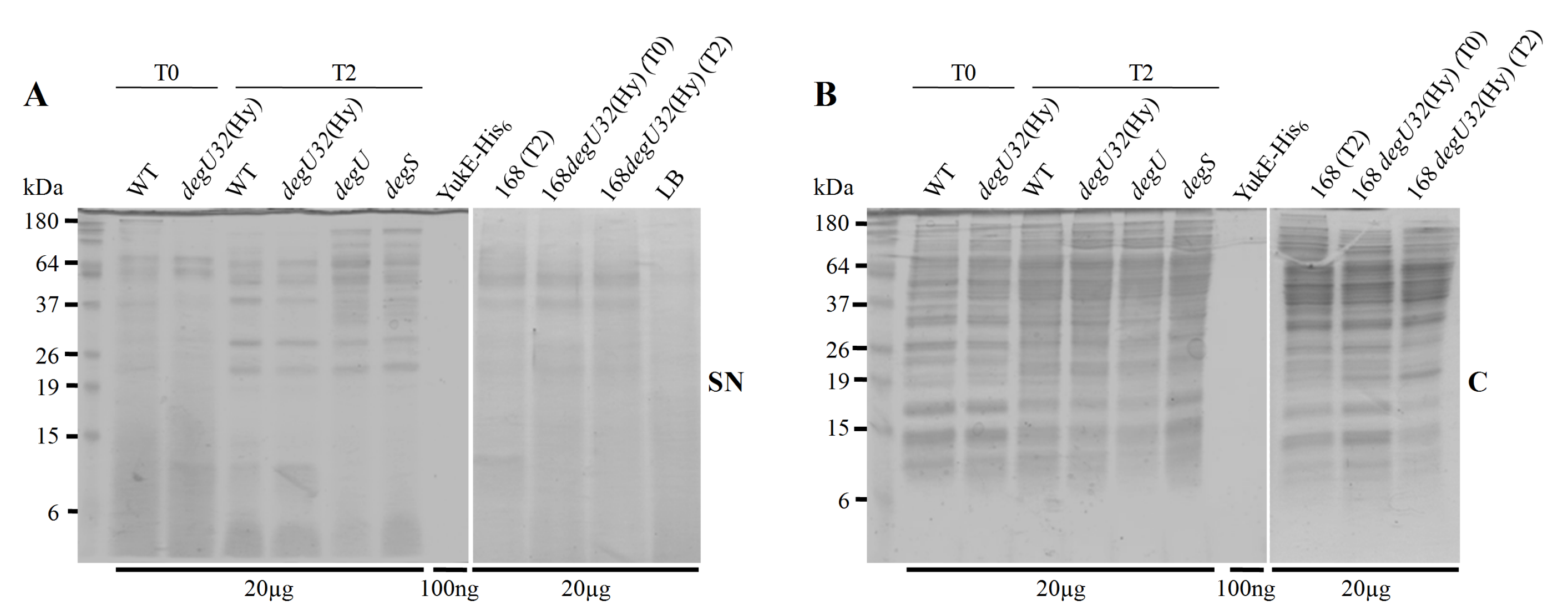

Supplement: Figure S1 — Coomassie blue-stained gels of the SN (A) and C (B) extracts subjected to Western blot analysis in Figure 3(B,C) (see text for details). Lane “LB” in panel A is to show the contribution of LB proteins to SN fractions. (TIF) [file pone.0067840.s001.tif]

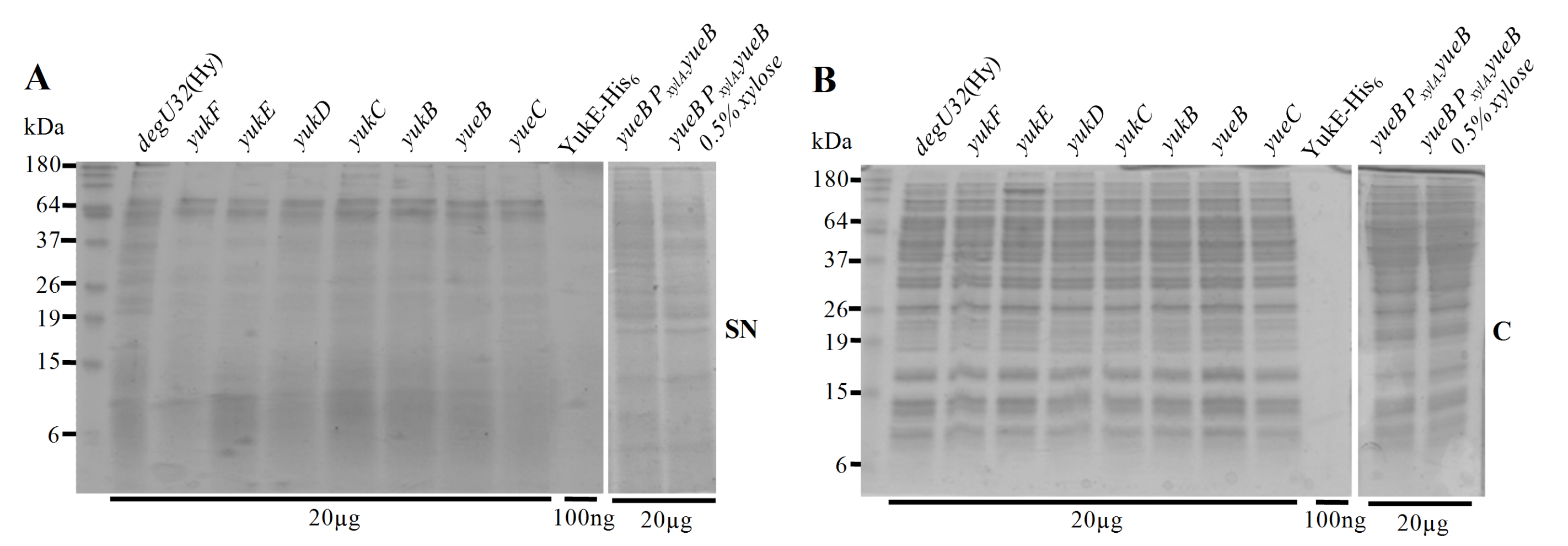

Supplement: Figure S2 — Coomassie blue-stained gels of the SN (A) and C (B) extracts subjected to Western blot analysis in Figure 5(A,B) (see text for details). (TIF) [file pone.0067840.s002.tif]
